# Biochemical diversity in *Allium* species: key metabolite profiles for breeding and bioprospecting

**DOI:** 10.3389/fpls.2025.1618572

**Published:** 2025-11-05

**Authors:** Ashwini Prashant Benke, Digambar Mokat, Vijay Mahajan

**Affiliations:** ^1^ Crop Improvement Division, ICAR-Directorate of Onion and Garlic Research, Rajgurunagar, Pune, India; ^2^ Department of Botany, Savitribai Phule Pune University, Pune, India

**Keywords:** phytochemical screening, flavonoids, phenolics, hierarchical clustering, principal component analysis, thiosulphinates content, antioxidant activity, functional food

## Abstract

The genus *Allium* encompasses a diverse range of species, including cultivated, wild, and underutilized varieties, each exhibiting significant biochemical diversity. This study aims to comprehensively analyze the phytochemical composition and biochemical variability in 19 *Allium* germplasm representing 16 species. The research focuses on key bioactive compounds such as flavonoids, phenolics, sulfur-containing compounds, and sugars to provide valuable insights for breeding programs, functional food development, and pharmaceutical applications. Biochemical profiling was conducted using standard assays (qualitative and quantitative) to determine antioxidant activity, thiosulfinate content, pyruvic acid levels, and sugar content. To analyze the data, hierarchical clustering analysis was performed to group the species, and Principal Component Analysis (PCA) was used to explain the total biochemical variance and differentiate the species based on their metabolic composition. The analysis revealed substantial variations across species. The average thiosulfinates content ranged from 5.33 to 26.12 µmol/g FW, total flavonoid content from 10.42 to 48.42 mg/100 g FW, and total phenolic content from 7.76 to 21.00 mg/100 g FW. The highest antioxidant activity (DPPH assay) was 6.05 µmol/g FW, while total sugar levels ranged from 0.51 to 8.79 g/100 g FW. The hierarchical clustering analysis grouped the *Allium* species into two major clusters: Cluster 1 (10 domesticated accessions) and Cluster 2 (9 wild and underutilized accessions). The clustering was driven primarily by thiosulfinate content, pyruvic acid levels, flavonoid content, and antioxidant activity, with wild species showing significantly higher concentrations of bioactive metabolites. The PCA explained 66.1% of the total biochemical variance, with PC1 contributing 38.5% and PC2 contributing 27.6%. Strong positive correlations were observed between total flavonoid content and antioxidant activity (r = 0.91, p < 0.001), total phenolic content and allicin (r = 0.87, p < 0.001), and total pyruvic acid and enzymatically produced pyruvic acid (r = 0.93, p < 0.001). This study underscores the significance of biochemical characterization in understanding the nutritional and medicinal potential of *Allium* species. The findings indicate a potential co-regulation of these biochemical pathways, as suggested by the strong positive correlations between key compounds. The results offer valuable insights for selecting superior *Allium* genotypes with enhanced bioactive properties, which is crucial for future interspecific *Allium* breeding programs and the development of new functional foods and pharmaceuticals.

## Introduction

The genus *Allium* is one of the largest monocotyledonous genera, comprising over 1,063 species distributed across temperate regions of the Northern Hemisphere (Anonymous, 2024). It includes economically and nutritionally important crops such as *Allium cepa* L. (onion), *A. sativum* L. (garlic), *A. fistulosum* L. (welsh onion), *A. tuberosum* Rotter ex Spreng. (Chinese chives), and *A. ampeloprasum* L. (leek). While cultivated *Allium* species form the backbone of vegetable production globally, their wild and underutilized counterparts represent a critical repository of genetic diversity (Fritsch and Friesen, 2002). Wild relatives of cultivated species serve as reservoirs of valuable traits, including enhanced resistance to diverse biotic and abiotic stresses, elevated concentrations of bioactive metabolites, and unique agronomic attributes, all of which hold significant potential for utilization in *Allium* improvement programs (Benke et al., 2024; Shukla et al., 2024).

India, recognized as a secondary center of diversity for the genus *Allium*, harbors a broad spectrum of cultivated, semi-domesticated, and wild species adapted to diverse agro-climatic zones. Within the genus, *A. cepa* (onion) and *A. sativum* (garlic) are the most widely cultivated and economically significant species. Other taxa, including *A. chinense* G. Don, *A. hookeri* Thwaites, *A. tuberosum*, and *A. fistulosum*, are distributed across the western Himalayas, northeastern states, and central India, where they are traditionally utilized as vegetables, condiments, and ethnomedicinal herbs (Devi et al., 2014; Pandey et al., 2020; Benke et al., 2022). ICAR-Directorate of Onion and Garlic Research act as National Active Germplasm site for *Allium* Species and holds approximate 2000 germplasm which includes 25 *Allium* species (Benke et al., 2024). India ranks second globally in onion production, contributing approximately 17 million tonnes annually (FAO, 2019), yet productivity remains constrained by climatic stresses and biotic challenges, despite the release of nearly 60 improved open pollinated varieties and a few hybrids from both public and private breeding programs (Krishna Kumar, 2015). In garlic, global production is overwhelmingly dominated by China (~23 MT; ≈75%), with India occupying the second position (~3 MT; ≈10%), where Madhya Pradesh alone contributes nearly two-thirds of the national output (Benke et al., 2024). Indian garlic exhibits marked variability in bulb morphology, clove characteristics, photoperiod requirement and pungency level, providing valuable opportunities for breeding, genetic improvement, and bioprospecting (Benke et al., 2022). Moreover, several semi-domesticated, underutilized and poorly characterized *Allium* species, particularly those endemic to the northeastern and Himalayan regions, remain largely unexplored (Negi et al., 2006; Verma et al., 2008), especially with respect to their biochemical composition and nutraceutical potential.

Indian research on biochemical profiling of onion, garlic and *Allium* species, such as *A. tuberosum* has demonstrated substantial variability in phenolics, flavonoids, and antioxidant activity among cultivars, with pronounced genotype × environment interactions influencing these traits (Lee et al., 2009; Dalamu et al., 2010; Khar et al., 2022; Kaur et al., 2024; Shukla et al., 2024; Benke et al., 2025) supported by complementary molecular analyses (Pasupula et al., 2024). Onion and garlic skins, in particular, have been identified as exceptionally rich in flavonoids and antioxidants, underscoring their nutraceutical potential and value as by-products (Sagar et al., 2022; Shabir et al., 2022; Raimo, 2020). More recent investigations on short-day Indian onion lines have employed multivariate approaches to associate biochemical diversity with agronomic traits such as bulb development, pungency, and storability (Benke et al., 2025; Gupta et al., 2024). Parallel studies have also explored the valorization of surplus and off-grade onions for antioxidant-rich extracts, promoting their use in functional food and bioeconomy applications (Abinaya et al., 2023). Several reviews have further emphasized the medicinal relevance of allicin and related thiosulfinates, consolidating their importance as functional metabolites with therapeutic potential (Verma et al., 2023; Singh et al., 2023).

Despite these advances, several gaps remain as biochemical research has been largely confined to cultivated onion and garlic, with limited exploration of wild and underutilized species that may harbor unique bioactive compounds and adaptive traits (Khar et al., 2022). The majority of studies have focused on a narrow set of biochemical parameters, such as phenolics, flavonoids, and antioxidant activity, while comprehensive metabolomic and transcriptomic investigations are still lacking. Furthermore, the strong influence of genotype × environment interactions on phytochemical expression has not been systematically addressed. Lack of standardized extraction methods and assay protocols also limits cross-comparability of results. Importantly, very few studies have attempted to integrate biochemical diversity with molecular markers (Pasupula et al., 2024; Benke et al., 2021) or genomic regions, leaving the genetic basis of phytochemical variation underexplored. Finally, the integration of biochemical traits into mainstream onion and garlic breeding programs remains limited, restricting their utilization in developing nutritionally enhanced and stress-resilient cultivars.

The variability within these metabolites across Indian *Allium* germplasm is not yet fully documented. Such biochemical diversity has implications not only for functional food development and pharmaceutical exploration but also for breeding programs targeting improved pungency, flavor, stress tolerance, and post-harvest qualities (Khar et al., 2022; Gonzalez-Morales et al., 2017). The absence of such comparative data restricts efforts to identify promising genotypes for introgression breeding or bioprospecting purposes. Moreover, wild and underutilized species despite their adaptation to diverse environments and high metabolite content remain underrepresented in mainstream *Allium* improvement efforts.

Biochemical profiling of *Allium* species is a powerful tool for exploring nutritional, functional, and therapeutic traits. Notably, the medicinal relevance of *Allium* species is closely linked to their organosulfur compounds, which exhibit antimicrobial, anticancer, antidiabetic, and cardioprotective activities (Arena et al., 2024; Rahman, 2007). Species within this genus are known to accumulate a wide range of phytochemicals, including flavonoids, polyphenols, saponins, sulfur-containing compounds (e.g., allicin, S-allyl cysteine), and sugars all of which contribute to flavor, aroma, antioxidant capacity, and health-promoting properties (Kothari et al., 2020; Seki et al., 2019). These compounds also serve as chemotaxonomic markers, facilitating the differentiation of closely related species and supporting evolutionary studies (Kamenetsky and Rabinowitch, 2001; Vukovic et al., 2021). GC-MS is a powerful technique for identifying and quantifying volatile sulfur-containing and other secondary metabolites in *Allium* species, but this study purposely focused on primary biochemical profiling of both volatile and non-volatile key metabolites such as total phenolics, flavonoids, allicin, pyruvic acid, sugars, and antioxidant activity using established, cost-effective, and reproducible spectrophotometric and colorimetric assays. These wet-chemical and enzymatic methods are particularly suitable for accurately measuring the targeted compounds, many of which are non-volatile and require simpler extraction and quantification protocols than GC-MS. Additionally, the use of GC-MS involves complex sample preparation like derivatization, which could introduce variability and reduce throughput, making it less feasible for large-scale comparative analysis across multiple species and accessions. The study’s scope was to generate foundational biochemical data focused on standardized markers relevant to nutrition and function, rather than exhaustive metabolite fingerprinting. Future research is planned to incorporate advanced metabolomics techniques, including GC-MS and LC-MS, to further characterize specific volatile and semi-volatile metabolites for detailed chemotaxonomic and functional insights among selected promising *Allium* lines.

In this context, the present study aims to systematically evaluate the biochemical diversity of 19 *Allium* germplasm representing 15 species, encompassing cultivated, semi-domesticated, and wild types which originated from different Indian agro-ecological regions and now adapted and acclimatized at ICAR-DOGR since last 10 years. The specific objectives are to: 1. Perform qualitative phytochemical screening to detect the presence of key secondary metabolites; 2. Quantify major bioactive compounds, including thiosulfinates (allicin), flavonoids, phenolics, antioxidant properties, sugars, carbohydrates and different forms of pyruvic acid; 3. Analyze interspecific variation and biochemical trait correlations using multivariate statistical methods such as hierarchical clustering and principal component analysis (PCA); 4. Identify metabolically rich unique species or accessions with potential applications in functional foods, medicinal formulations, and crop improvement. Therefore, by establishing a biochemical baseline, this study aims to bridge critical knowledge gaps and support the conservation, utilization, and genetic enhancement of *Allium* species present in India.

## Materials and methods

**Field site**: This experiment was carried out at the *Allium* germplasm collection center, situated at 27°19′00.2″ N latitude, 82°25′00.1″ E longitude, and an elevation of 553.8 m above sea level, within the Indian Council of Agricultural Research (ICAR) – Directorate of Onion and Garlic Research (DOGR), Pune, India. According to meteorological data from the Indian Meteorological Department (IMD) and the ICAR-DOGR meteorology laboratory, the region experiences a cold semiarid climate, characterized by an average annual rainfall of 251.7 mm, an average temperature of 21.4°C, and a relative humidity of 48%. Experiment was carried out during winter 2023.

**Material**: The selection of *Allium* germplasm for this study was strategically designed to capture maximum biochemical and taxonomic diversity within the genus. A total of 19 accessions representing 15 species (as detailed in **Table 1**) were chosen to include both widely cultivated types, such as *A. cepa, A. sativum* and *A. fistulosum*, *A. cepa aggregatum alongside lesser-studied wild and underutilized* sp*ecies including A. hookeri, A. chinense, A. alticum, A. macranthum, A tuberosum, A. angulosum, A. schoenoprasum, A. acalonicum, A. ampeloprasum, A. ledeborianum* and *A. przewalskianum*. These accessions represent a broad phylogenetic spectrum and encompass different stages of domestication and justified by their agro-morphological and molecular screening (Benke et al., 2024). The chosen germplasm also included species previously identified to exhibit distinct morphological features, stress resilience, or notable agronomic traits (Benke et al., 2024), aligning with ongoing breeding and conservation efforts. To maintain uniform growth conditions and enable valid comparative analysis, 10 bulbs or rhizomes per accession were cultivated at a spacing of 0.3 × 0.3 m under standardized agronomic practices including regular irrigation, weeding, and top dressing at the ICAR-Directorate of Onion and Garlic Research (ICAR-DOGR), Pune. This inclusive and methodical selection and cultivation approach ensured comprehensive representation of natural biochemical variability within Indian *Allium* species, providing a solid foundation for subsequent phytochemical and antioxidant profiling.

**Table 1:** Collection, ecological status and source or origin of 19 *Allium* germplasm including *A. cepa, A. sativum* and other underutilized wild *Allium* species used for biochemical diversity studies.

S. No	*Allium* species	Collector / IC / EC No.	Ecological status	Source/ Origin of collection
1	*A. fistulosum L.(HP)*	CGN – 16418/ AKO - 1 CHINA	Endemic	Centre for Genetic Resource, Netherland
2	*A. cepa agr -* 5 manipur	–	Native	Manipur, India
3	*A. przewalskianum*	NMK -117	Native	Manipur, India
4	*A. hookeri*	NMK - 3235	Native	Manipur, India
5	*A. schoenoprasum*	NR – 6/ NGB -14574	Native	Sikkim, India
6	*A. altaicum pall*	EC - 328485	Native	ICAR-NBPGR, New Delhi
7	*A. ledeborianum*	EC - 328491	Native	ICAR-NBPGR, New Delhi
8	*A. chinense*	Chollang White (Rakkyo)	Native	Sikkim, India
9	*A. macranthum*	NMK - 3240	Native	Manipur, India
10	*A. schoenoprasum*	NR -6/NGB 5469	Native	Sikkim, India
11	*A. ampeloprasum*	NMK- 3211	Native	Manipur, India
12	*A. angulosum*	EC - 328486	Native	ICAR-NBPGR, New Delhi
13	*A. tuberosum*	NMK -3231 (NF)	Native	Manipur, India
14	*A. ascalonicum*	Pran	Native	ICAR-NBPGR, New Delhi
15	*A. sativum* var. Bhima Omkar	IC-569789	Native	ICAR-DOGR, Pune
16	*A. sativum* var. Bhima Purple	IC-570742	Native	ICAR-DOGR, Pune
17	*A. cepa var.* Bhima Super	IC-561259	Native	ICAR-DOGR, Pune
18	*A. cepa var.* Bhima Shweta	IC-572761	Native	ICAR-DOGR, Pune
19	*A. cepa var.* Bhima Kiran	IC-572766	Native	ICAR-DOGR, Pune

Abbreviations: Cac: Albumin-corrected calcium. P: phosphate. ALP: alkaline phosphatase. TmP/GFR: ratio of tubular maximal reabsorption of phosphate (TmP) to glomerular filtration rate (GFR). FGF23: fibroblast growth factor 23. 25-OH vit D: 25-hydroxy vitamin D. 1,25(OH)2 vit D: 1,25 dihydroxy vitamin D. PTH : parathormone. -: not assessed.

## Methods

### Phytochemical analysis of *Allium* germplasm

#### Preparation of extract

Fresh *Allium* leaves were collected, thoroughly washed with distilled water (DW) to remove dirt and contaminants, and used for extraction. The extraction was carried out using two different solvents DW and 70% ethanol to ensure the efficient isolation of both polar and semi-polar phytochemicals. This dual-solvent extraction method enables the efficient isolation of a broad range of phytochemicals. In qualitative phytochemical screening, distilled water (DW) primarily extracts hydrophilic compounds, whereas 70% methanol extracts a broader and more diverse profile of phytochemicals, especially medium-polar compounds like flavonoids and saponins. Therefore, using both solvents provides complementary information, enhancing the overall phytochemical coverage and detection accuracy. The presence of a compound in the test was recorded as ‘Y,’ while its absence was marked as ‘X.’ Tests that did not yield a clear result may be due to low concentration of targeted compound or solvent or extraction method may not efficiently solubilize the compound of interest due to polarity issues were labeled as ‘not applicable’.

For aqueous extraction, 10 g of fresh *Allium* leaves was soaked in 100 mL of distilled water in a conical flask and left for 24 hours at room temperature with occasional stirring. The extract was then filtered using Whatman No. 1 filter paper, concentrated by water bath (40–50°C) or freeze-dried, and stored at 4°C. For ethanolic extraction, the same procedure was followed using 70% ethanol. Phytochemical tests were performed on both extracts across 19 *Allium* germplasm. Each assay was conducted in triplicate, with either distilled water or the respective solvent used as a control, depending on the test requirements. Additionally, three variable concentrations were employed to generate the standard calibration curve. The details of test as below.

#### Test for carbohydrates (Molisch’s test)

Two milliliters (2 mL) of the extract was placed in a test tube, followed by the addition of two drops of Molisch’s reagent. Concentrated sulfuric acid (H_2_SO_4_) was carefully added along the walls of the test tube to form a distinct layer. The appearance of a purple or reddish-violet ring at the interface confirms the presence of carbohydrates (Harborne, 1998).

#### Test for proteins (Biuret test)

To detect the presence of proteins, two qualitative tests were conducted using specific reagents. In the Biuret test, an equal volume of Biuret reagent, consisting of sodium hydroxide and copper sulfate, was added to 2 mL of extract. The formation of a violet or purple coloration indicated the presence of proteins. Similarly, in Millon’s test, a few drops of Millon’s reagent were added to 2 mL of the extract, followed by gentle heating. The appearance of a red or brick-red coloration confirmed the presence of proteins, further validating the result. These tests collectively provide strong evidence of protein content in the sample (Sadasivam and Manickam, 2008).

#### Test for starch (Iodine test)

A few drops of iodine solution (I_2_-KI solution) were added to 2 ml extract and mixed gently. The appearance of a blue-black coloration indicates the presence of starch (Sadasivam and Manickam, 2008).

#### Test for phenolic compounds and tannins

Three milliliters (3 mL) of extracts were tested with 3% ferric chloride (FeCl_3_). A dark green coloration confirms the presence of phenolic compounds. Additionally, 2 mL of the extract was treated with a few drops of 10% ferric chloride solution. The appearance of a blue-black or blue-green precipitate indicates the presence of tannins (Oladeji et al., 2020).

#### Test for flavonoids

Two milliliters (2 mL) of dilute sodium hydroxide (NaOH) was added to 3 mL of the extract in a test tube. The development of a yellow solution, which turns colorless upon the addition of concentrated hydrochloric acid (HCl), indicates the presence of flavonoids (Bhat, 2020).

#### Test for saponins

Three milliliters (3 mL) of the extract was diluted with 20 mL of distilled water and vigorously shaken in a graduated cylinder for 15 minutes. The formation of a stable foam layer approximately 1 cm in height confirms the presence of saponins (Arulmozhi et al., 2018).

#### Test for anthraquinones (Borntrager’s test)

Three milliliters (3 mL) of extract was mixed with 3mL of benzene in a test tube and shaken well. A few drops of ammonia solution (10%) were added to the mixture. The formation of a pink, red, or violet coloration in the ammoniacal (upper) layer confirms the presence of anthraquinones (Harborne, 1998).

#### Test for alkaloids

To confirm the presence of alkaloids, a series of qualitative tests were conducted using specific reagents, each producing characteristic precipitates. Mayer’s test involved the addition of Mayer’s reagent (potassium mercuric iodide solution) to the extract, leading to the formation of a white or creamy precipitate, indicative of alkaloids. Similarly, in Dragendorff’s test, the extract was treated with Dragendorff’s reagent (potassium bismuth iodide solution), resulting in a distinct orange or reddish-brown precipitate, further affirming alkaloid presence. Wagner’s test was performed by adding Wagner’s reagent (iodine in potassium iodide solution) to the extract, which produced a reddish-brown precipitate, serving as another positive confirmation. Lastly, in Hager’s test, the extract was mixed with Hager’s reagent (saturated picric acid solution), leading to the formation of a yellow precipitate, reinforcing the presence of alkaloids. These complementary tests provide a robust indication of alkaloids in the sample, ensuring the accuracy and reliability of the phytochemical screening (Harborne, 1998).

#### Test for glycosides

A small portion of the extract was placed in a test tube, followed by the addition of 2 mL of glacial acetic acid, a drop of ferric chloride solution, and 2 mL of concentrated sulfuric acid (H_2_SO_4_). The appearance of a distinct brown ring at the interphase confirms the presence of glycosides (Arulmozhi et al., 2018).

#### Test for steroids

A few drops of concentrated sulfuric acid (H_2_SO_4_) were added to a small portion of the aqueous extract in a test tube and gently shaken. The formation of a red coloration indicates the presence of steroids (Bhat, 2020).

#### Test for amino acids

To confirm the presence of amino acids, qualitative tests were performed using specific reagents. In the Ninhydrin test, two milliliters (2 mL) of the aqueous extract were treated with a few drops of 0.2% Ninhydrin reagent and heated in a water bath for a few minutes. The development of a purple or blue-violet coloration indicated the presence of free amino acids.

### Quantitative biochemical traits

In quantitative analysis, data were recorded for nine quantitative biochemical parameters, including thiosulfinate content (µmol/g fresh weight (FW)), antioxidant capacity measured as DPPH AE (µmol/g FW) and FRAB (mg/ml), total flavonoid content (TFC) expressed in QE (mg/g FW), total phenolic content (TPC) in GE (mg/g FW), total pyruvic acid (TPA), basal pyruvic acid (BPA), enzymatically produced pyruvic acid (EPA)(µmol/gm FW), total sugar content (g/100g FW), and reducing sugar content (g/100g FW). Each parameter was analyzed in triplicate to ensure accuracy and reliability. Each parameter was analyzed separately, with three independent biological replicates performed for statistical robustness. The detailed methodology for each parameter is provided below.

#### Pyruvic acid

The estimation of pyruvic acid is performed using the TCA (Trichloroacetic Acid) method, which involves DNPH (2,4-dinitrophenylhydrazine) reaction to form a hydrazone complex measured at 420 nm using a spectrophotometer (Schwimmer and Weston, 1961). In brief, 1 gm of the sample paste is mixed with 3 ml of water, vortexes, and filtered. From the filtrate, 1 ml is diluted with 19 ml distilled water for onion and 15 ml for garlic. Then, 1 ml of this diluted sample is mixed with 1 ml DW and 1 ml DNPH (0.63 mM). The reaction mixture is incubated in a 37°C water bath for 10 minutes, followed by the addition of 5 ml of 0.6N NaOH. The mixture is kept in the dark for 5 minutes, and absorbance is measured at 420 nm. For the control, 1 gm of the sample is treated with 3 ml of 5% TCA, incubated at 37°C for 1 hour, and then filtered and the procedure is repeated as in the sample preparation. The blank consists of 2 ml DW and 1 ml DNPH, serving as a baseline reference. The results were expressed as total pyruvic acid which refers to the overall amount of pyruvic acid present in the sample, including both the free pyruvic acid and the pyruvic acid formed enzymatically from precursor compounds., basal pyruvic acid which represents the naturally occurring pyruvic acid present in the sample before enzymatic conversion of precursors *viz*. S-alk(en)yl-L-cysteine sulfoxides (ACSOs), and enzymatically produced pyruvic acid. This refers to the pyruvic acid generated by enzymatic action, primarily by alliinase in garlic and onions, which breaks down sulfur-containing compounds into pyruvic acid.

#### Average allicin or total thiosulfinate content

A 1-gram sample of paste was mixed with 15 mL of HEPES buffer and centrifuged. To 80 µL of the resulting supernatant, 120 µL of L-cysteine was added, and the mixture was incubated at 37°C for 15–20 minutes. Subsequently, 300 µL of HEPES buffer and 100 µL of DTNB were introduced. Absorbance was measured at 412 nm using a Tecan Infinite 200 Pro M Nano+ spectrophotometer (Männedorf, Switzerland), with 200 µL of the sample placed in a plate (Cantwell et al., 2003).

#### Total phenolic content

The Folin Ciocalteu method (Singleton et al., 1999) was used to determine TPC. A total of 100 µL of Folin Ciocalteu reagent was added to 200 µL of sample extract, vortexed for a minute, and incubated for 5 minutes. Then, 800 µL of 700 mM sodium carbonate (Na_2_CO_3_) was added, and the mixture was incubated for 120 minutes in the dark. The sample (200 µL) was loaded into a microtitration plate, and absorbance was recorded at 765 nm using a Tecan Infinite 200 Pro M Nano+ spectrophotometer. The results were quantified using a Gallic acid standard curve (y = 3.8765x + 0.0127, R² = 0.9998) and expressed as mg GAE/g FW.

#### Total flavonoid content

The aluminum chloride colorimetric assay (Sembiring et al., 2018, with modifications) was used to determine TFC. Briefly, 100 µL of sample extract was mixed with 400 µL of distilled water, 300 µL of 10% aluminum chloride (AlCl_3_), and 300 µL of 5% sodium nitrite (NaNO_2_). After 5 minutes of incubation, 200 µL of 1N sodium hydroxide (NaOH) and 240 µL of distilled water were added. The absorbance was measured at 510 nm (Kumaran and Karunakaran, 2007). The results were quantified using a quercetin standard curve (y = 7.1021x − 0.005, R² = 0.9999) and expressed as mg QE/g FW.

#### Total antioxidant capacity (DPPH assay)

The DPPH (1,1-diphenyl-2-picrylhydrazyl) assay (Nuutila et al., 2003) was performed by mixing 100 µL of sample extract with 390 µL of 0.1 mM DPPH solution. The mixture was incubated in the dark at 25°C for 30 minutes, and absorbance was recorded at 515 nm using a Tecan Infinite 200 Pro M Nano+ spectrophotometer. The results were determined using a Trolox standard curve (y = −21.0864x + 21.2202, R² = 0.9999) and expressed as µmol TE/g FW.

#### Total antioxidant capacity (FRAB assay)

The Ferric Reducing Antioxidant Power (FRAP) assay was carried out following Benzie and Strain (1996) with minor modifications. Briefly, 100 µL of sample extract was mixed with 300 µL of freshly prepared FRAP reagent (300 mM acetate buffer, pH 3.6; 10 mM TPTZ solution in 40 mM HCl; and 20 mM FeCl_3_·6H_2_O in a 10:1:1 ratio). The reaction mixture was incubated at 37°C for 30 minutes, and absorbance was measured at 593 nm using a Tecan Infinite 200 Pro M Nano+ spectrophotometer. A standard curve (y = 0.0047x + 0.0316, R² = 0.9985) was used to quantify antioxidant activity, and results were expressed as mg/ml FW.

#### Total sugar content

A 50 µL extract was diluted with 450 µL of distilled water, followed by the addition of 500 µL of phenol and 500 µL of sulfuric acid. The mixture was incubated at room temperature for 15 minutes, and absorbance was recorded at 480–490 nm using a Tecan Infinite 200 Pro M Nano+ spectrophotometer. The results were expressed as g/100 g FW, following the Lane and Eynon method (Ranganna) (Mathew and Abraham, 2006).

#### Reducing sugar content

To 100 µL of sample extract, 100 µL of DNS reagent was added, and the mixture was boiled for 5 minutes. Absorbance was recorded at 540 nm using a Tecan Infinite 200 Pro M Nano+ spectrophotometer. The results were expressed as g/100 g FW (Mathew and Abraham, 2006).

### Chemicals and reagents

All chemicals used for biochemical analysis were of analytical grade and procured from Thermo Fisher Scientific, Mumbai, India. Additionally, high-purity analytical reagents were sourced from Sigma-Aldrich, Mumbai, India, ensuring precision and reliability in experimental procedures.

### Statistical analysis

Means and standard errors were calculated for all variables for each species using SAS 9.3, GLM Proc. In addition, cluster analysis, principal component analysis and correlation analysis of the quantitative biochemical traits of the 19 *Allium* germplasm was estimated by considering a bootstrap analysis with three replications and the tree was constructed by using hierarchical clustering based on Ward (1963) method by JMP 10.3 Pro (by SAS 9.3 base). The correlation graph was constructed using R studio software with package (library (Hmisc) specifically rcorr (as.matrix (mtcars [, c (“mpg”, “cyl”, “disp”, “hp”)]))).

## Results

### Phytochemical screening of *Allium* species

The genus *Allium* (onion, garlic, chives, and related species) is rich in bioactive compounds such as organosulfur metabolites, phenolics, flavonoids, saponins, and sterols, which contribute to antioxidant, antimicrobial, anti-inflammatory, and other health-promoting activities, making them valuable for bioprospecting. Qualitative phytochemical screening provides a quick and cost-effective means to detect these metabolites, with solvent choice (aqueous vs. hydroalcoholic) influencing recovery of polar and semi-polar compounds.

In results, a qualitative phytochemical analysis was conducted on 19 *Allium* germplasm representing 15 *Allium* species. The presence (Y) or absence (X) of key biochemical compounds was assessed across multiple biochemical tests. The phytochemical analysis of various *Allium* species using distilled water and 80% methanol as a solvent revealed the presence of several polar and semi-polar bioactive compounds (**Supplementary Tables S1**, **S2**). Carbohydrates were found to be consistently present across all tested species, indicating their fundamental role in these plants. Proteins were not strongly detected by the qualitative assays (NaOH-CuSO_4_ and Millon’s tests), suggesting their occurrence in relatively low concentrations or limitations of these colorimetric tests in *Allium* extracts. Similarly, starch was not detected in any of the species.

Secondary metabolites such as phenols, flavonoids, and flavonols were present in all species, highlighting their potential antioxidant properties. Additionally, saponins were found in every species tested using ethanol solvent, suggesting their possible role in plant defense mechanisms and medicinal properties (**Supplementary Table S2**). Interestingly, tannins were absent in all samples, implying that these species may have a lower astringency compared to other plants rich in tannins. The screening for alkaloids, using Mayer’s, Dragendorff’s, Wagner’s, and Hager’s tests, yielded negative results for all species, indicating the presence of these nitrogenous compounds in trace amount. Similarly, glycosides were mostly absent, except for a positive result in the Salkowski test, suggesting the presence of steroidal or terpenoid glycosides in all species.

Furthermore, the presence of amino acids across all species suggests that these *Allium* plants could serve as a source of essential nutrients. Fats were not specifically analyzed in this study. Overall, this phytochemical investigation confirms that *Allium* species are rich in carbohydrates, phenols, flavonoids, saponins, glycosides (as indicated by the Salkowski test), and amino acids, while proteins, starch, tannins, and alkaloids were present in trace amount. These findings contribute to the understanding of the medicinal and nutritional potential of *Allium* species, supporting their use in traditional and modern bioprospecting applications.

### Analysis of biochemical parameters

Standard assays enable broad profiling of key constituents, including characteristic sulfur-rich compounds, thereby facilitating the assessment of phytochemical diversity in *Allium* germplasm. The descriptive statistics for twelve biochemical traits in the evaluated *Allium* germplasm revealed pronounced phenotypic variability (**Table 2**). Mean values spanned from 0.50 µmol g^−1^ FW for enzymatically produced pyruvic acid to 36.70% for carbohydrate content. Among phytochemicals, total flavonoid content (23.06 mg 100 g^−1^ FW) and total phenol content (22.22 mg 100 g^−1^ FW) were most abundant, exhibiting wide ranges of 10.42–48.42 mg 100 g^−1^ FW and 14.35–38.47 mg 100 g^−1^ FW, respectively. The highest coefficients of variation (CV) were observed for enzymatically produced pyruvic acid (35.72%), total antioxidant capacity (FRAP assay) (29.67%), and basal pyruvic acid (28.26%), indicating substantial metabolic heterogeneity. Variance was greatest for total flavonoid content (143.66), whereas carbohydrate content showed the highest absolute mean with comparatively low variability (CV = 16.03%). Reducing sugar content ranged from 0.51 to 8.79 g 100 g^−1^ FW (CV = 25.48%), while protein content varied between 0.36 and 1.99 mg ml^−1^ (CV = 20.68%). These results highlight considerable biochemical diversity, likely reflecting both genotypic differences and environmental influences, and identify key traits of interest for genetic improvement and bioprospecting (**Table 3**).

**Table 2:** Mean and related statistical parameters of twelve biochemical traits recorded on 19 Allium germplasm representing 15 Allium species.

Parameters	Average Allicin TOT Thio (µmol/g FW)	Total Antioxidant capacity (μmol/g FW) DPPH assay	Total Antioxidant capacity (mg/ml)FRAB Assay	Total Flavanoid Content QE (mg/100 g FW)	Total Phenol content GE (mg/100 g FW)	Total Pyruvic Acid (µmol/g FW)	Basal Pyruvic Acid (µmol/g FW)	Enzymatically Produced Pyruvic Acid (µmol/g FW)	Reducing Sugar ( g/100g FW)	Total Sugar ( g/100g FW)	Carbohydrate (%)	Protein (mg/ml)
Mean	14.50	3.40	6.73	23.06	22.22	15.04	12.81	2.23	0.50	3.35	36.70	1.13
Std. Dev	5.20	0.69	2.67	11.99	6.10	3.89	3.62	1.47	0.48	2.19	13.22	0.35
Std Err	1.19	0.16	0.61	2.75	1.40	0.89	0.83	0.34	0.11	0.50	3.03	0.08
CV	25.89	20.32	29.67	21.98	27.47	25.87	28.26	25.83	35.72	25.48	16.03	20.68
Min (average)	5.33	2.09	3.71	10.42	14.35	7.76	6.32	0.22	0.04	0.51	11.30	0.36
Max (average)	26.12	4.02	12.87	48.42	38.47	21.00	18.61	6.05	1.74	8.79	66.90	1.99
Variance	27.08	0.48	7.12	143.66	37.26	15.13	13.10	2.16	0.23	4.80	174.86	0.12

**Table 3:** Promising biochemical traits identified in cultivated, wild, and underutilized Allium species evaluated in the study..

Promising Species	Promising Traits
*A. cepa (Onion)*	High carbohydrates and sugars; clustered with cultivated types
*A. sativum (Garlic)*	High carbohydrates and sugars; widely used nutraceutical source
*A. fistulosum (Welsh onion)*	Moderate biochemical diversity; cultivated type
*A. cepa var. aggregatum (Shallot)*	Carbohydrate-rich; clustered with cultivated onion
*A. hookeri*	High allicin, pyruvic acid, flavonoids, phenolics, antioxidant activity
*A. macranthum*	Enriched in allicin, flavonoids, phenolics, antioxidant capacity
*A. tuberosum (Chinese chives)*	Unique organosulfur and antioxidant profiles; underutilized species
*A. chinense (Chinese onion)*	Distinct biochemical traits; moderate antioxidants
*A. schoenoprasum (Chives)*	Elevated antioxidant potential; moderate phenolic content
*A. przewalskianum*	Stress resilience; unique phytochemical richness

Notes: Comparisons were made within the same month group under the same indicator, and there were no significant differences between data with the same lowercase letter (p<0.05). SGI represents simulated nibbling intensities.

### Cluster analysis

#### Hierarchical clustering and biochemical variability in *Allium* germplasm

**Figure 1:**
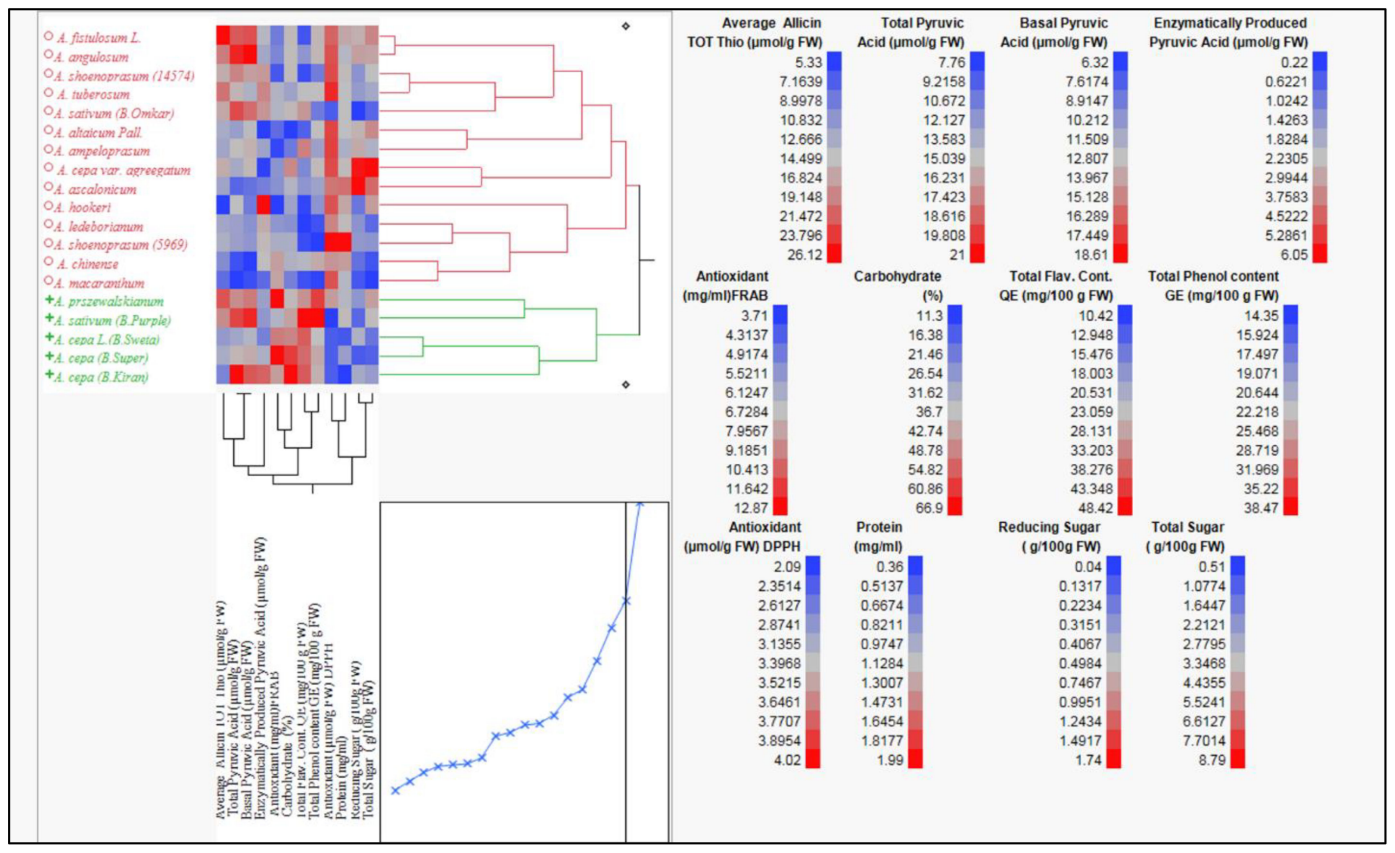
Hierarchical clustering and heatmap visualization of 15 Allium species representing 19 Allium germplasm based on genetic distance among 12 biochemical traits including thiosulfinate content, pyruvic acid (total, basal, enzymatically produced), flavonoid content, phenol content, antioxidant activity (DPPH and FRAB Assay), reducing sugar, total sugar and protein extracted using ward (1963) method. The color scale indicates relative concentrations, and dendrogram show species clustering based on metabolite similarity showing two major groups:1 cluster contain domesticated species; 2 cluster contain underutilized and wild Allium species. (4).

Clustering of *Allium* species based on their biochemical profiles will provide a powerful tool to decipher chemotypic diversity and identify patterns of similarity among accessions. This approach enables the targeted selection of genotypes rich in specific bioactive compounds, supporting breeding programs aimed at enhancing nutritional and therapeutic traits. It will also facilitates the conservation of valuable genetic resources and prioritizes accessions for functional food, nutraceutical, and pharmaceutical development. Hierarchical clustering analysis based on genetic distance of twelve biochemical parameters (**Figure 1**; **Supplementary Tables S3**, **S4**) grouped 15 *Allium* species, comprising 19 germplasm accessions, into two major clusters. The first cluster (red branch) predominantly included cultivated species such as *A. cepa* (including bulb types and local varieties) and *A. sativum* accessions. The second cluster (green branch) was largely composed of wild and underutilized species, including *A. hookeri*, *A. tuberosum*, *A. chinense*, and *A. macranthum*. The clear separation between clusters reflects substantial biochemical divergence between domesticated and wild taxa.

The heatmap adjacent to the dendrogram visualizes standardized (z-score) values (**Figure 1**) for traits including total thiosulfinate content, total pyruvic acid, basal pyruvic acid, enzymatically produced pyruvic acid, total antioxidant capacity (FRAP and DPPH assays), total flavonoid content, total phenol content, carbohydrate percentage, reducing sugar, total sugar, and protein content. Distinct color patterns differentiate the two main clusters, with wild species generally exhibiting higher levels of allicin, pyruvic acid, flavonoids, and phenolics, while cultivated onions tended to show higher carbohydrate and sugar content.

Trait-specific heat maps (right panel) highlight the range of variation across germplasm. Allicin levels varied from 5.33 to 26.12 µmol g^−1^ FW, with the upper range dominated by wild accessions. Total flavonoid content ranged from 10.42 to 48.42 mg 100 g^−1^ FW, and total phenol content from 14.35 to 38.47 mg 100 g^−1^ FW, with wild accessions frequently at the higher end. Enzymatically produced pyruvic acid ranged 0.22 to 6.05 µmol g^−1^ FW, and total sugar content ranged from 0.51 to 8.79 g 100 g^−1^ FW, with cultivated types generally richer in sugars. Antioxidant capacity, as measured by DPPH and FRAP assays, also showed marked differences, with wild species often surpassing cultivated onions.

The clustering pattern was largely influenced by allicin, pyruvic acid (total, basal, and enzymatically produced), antioxidant activity, and sugar-related traits. These findings suggest that wild *Allium* species harbor elevated concentrations of health-promoting compounds, offering valuable genetic resources for breeding programs targeting enhanced nutritional quality, stress tolerance, and disease resistance in cultivated onions and garlic.

#### Correlation among biochemical traits in *Allium* germplasm

**Figure 2:**
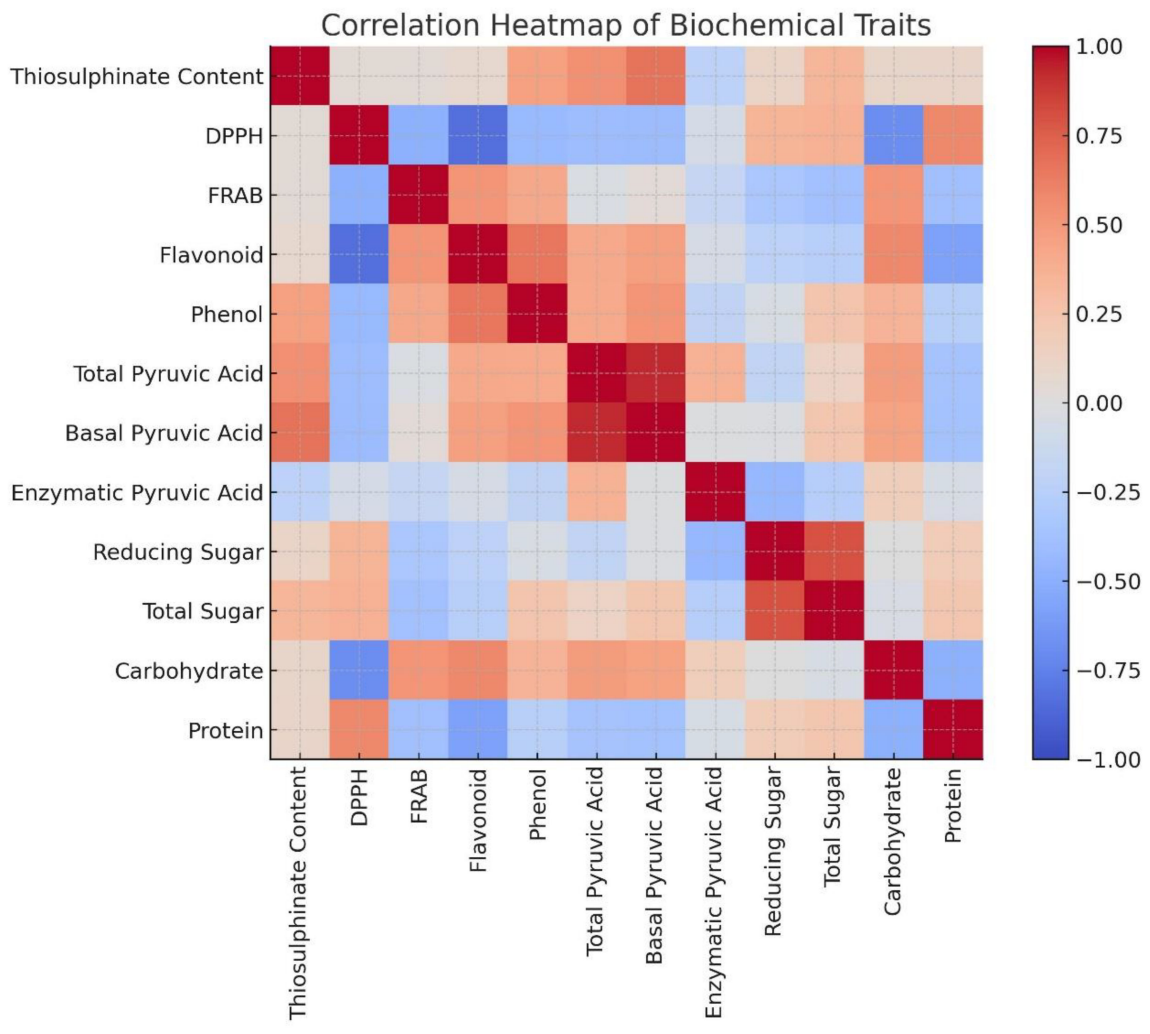
Correlation heatmap depicting the pairwise relationships among twelve key biochemical traits assessed in 19 *Allium* germplasm accessions representing 15 distinct *Allium* species. The heatmap visualizes the strength and direction of correlations, where dark red indicates a strong positive correlation (r ≈ +1), dark blue indicates a strong negative correlation (r ≈ –1), and white reflects little to no correlation (r ≈ 0). The figure was generated using R programming language, employing a combination of packages including ggplot2, heatmap, corrplot, and Complex Heatmap to enhance visualization and interpretability of trait interrelationships.

Correlation studies will help to reveal how different metabolites interact or co-vary within a genotype or across germplasm, providing insights into metabolic pathways and trait interdependence. Analyzing these correlations aids in identifying key traits for selection in breeding programs, facilitates the prioritization of accessions for functional food or pharmaceutical use, and enhances the understanding of biochemical diversity within *Allium* germplasm. Pearson’s correlation analysis (**Table 4**) revealed several significant associations among the twelve biochemical traits. Strong positive correlations were observed between total pyruvic acid and basal pyruvic acid (r = 0.9262), and between allicin content and basal pyruvic acid (r = 0.6671), indicating that pungency-related sulfur compounds are closely interlinked. Total phenol content was moderately correlated with both total flavonoid content (r = 0.6504) and basal pyruvic acid (r = 0.5121), suggesting a partial overlap in the biochemical pathways contributing to phenolic and pungency traits. Carbohydrate percentage showed a positive association with total flavonoid content (r = 0.5759) and FRAP-based antioxidant capacity (r = 0.5170), whereas protein content was positively related to DPPH-based antioxidant activity (r = 0.5717) but negatively associated with carbohydrate percentage (r = –0.4913). Reducing sugar exhibited a strong positive correlation with total sugar (r = 0.7969), reflecting shared carbohydrate metabolism, but was negatively correlated with enzymatically produced pyruvic acid (r = –0.4452) (**Figure 2**).

Notably, DPPH-based antioxidant capacity was negatively correlated with total flavonoid content (r = –0.8302) and carbohydrate percentage (r = –0.6868), suggesting that radical scavenging efficiency in *Allium* may be influenced by other antioxidant constituents beyond flavonoids. Similarly, FRAP-based antioxidant capacity showed a negative relationship with DPPH-based antioxidant activity (r = –0.4871), implying differences in antioxidant profiles captured by the two assays. These correlations indicate that certain biochemical traits are tightly co-regulated (e.g., sulfur compounds and pungency indices), while others exhibit trade-offs (e.g., antioxidant activity vs. carbohydrate accumulation). Such patterns provide insight into the biochemical network structure in *Allium* and can guide selection strategies in breeding programs targeting multiple quality traits. These correlation patterns highlight biochemical interdependencies that are relevant for the interpretation of principal component analysis results.

#### Principal Component Analysis (PCA) of Biochemical trait of *Allium* species

The Kaiser–Meyer–Olkin (KMO) test measures sampling adequacy for PCA by evaluating whether the variables have enough common variance. In *Allium* biochemical studies, a high KMO value indicates that the dataset is suitable for dimension reduction, ensuring reliable identification of patterns and relationships among biochemical traits. The (KMO) measure of sampling adequacy yielded an overall value of 0.65, indicating a moderate suitability of the biochemical dataset for multivariate analysis. Most biochemical traits, including allicin content, total flavonoid content, total phenol content, total pyruvic acid, basal pyruvic acid, enzymatically produced pyruvic acid, reducing sugar, total sugar, carbohydrate, and protein, exhibited individual MSA values of 0.60, suggesting acceptable shared variance. However, antioxidant activity measured by DPPH and FRAP showed notably low MSA values (0.06), implying limited correlation with other variables. Bartlett’s test assesses whether the correlation matrix significantly differs from an identity matrix, confirming that variables are interrelated and suitable for multivariate analysis. A significant result ensures that PCA can meaningfully reduce data complexity without losing essential information about trait associations. Bartlett’s test of sphericity was highly significant (χ² = 362.49, df = 11, *p* < 2.2 × 10^−16^), confirming that the correlation matrix was not an identity matrix and that the dataset had sufficient inter-variable relationships for factor analysis. Together, KMO and Bartlett’s tests validated the suitability of data for PCA, while PCA itself provides a robust framework for summarizing complex biochemical datasets and uncovering meaningful patterns in *Allium* germplasm.

**Figure 3:**
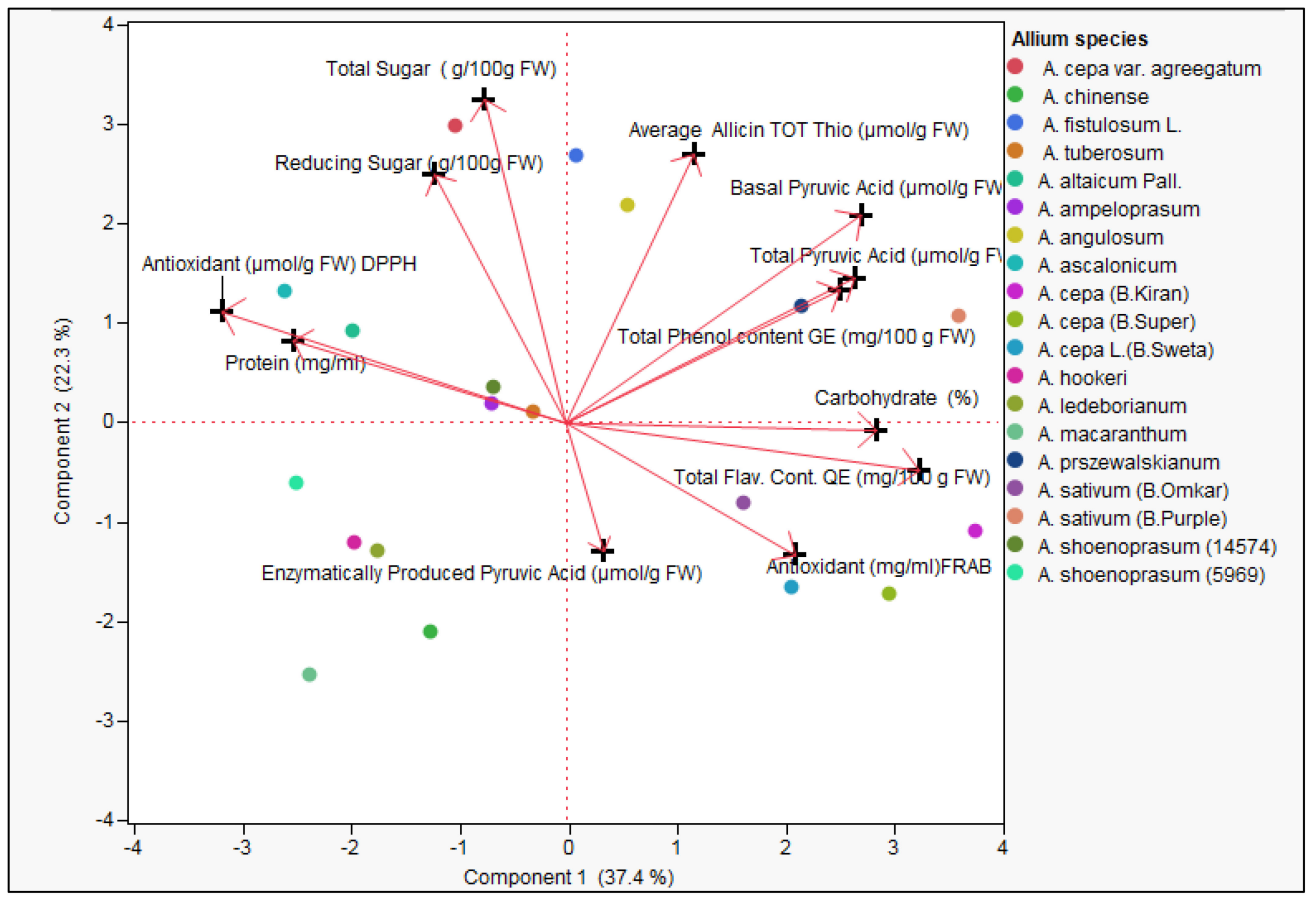
Principal Component Analysis (PCA) biplot representing the distribution of 19 Allium germplasm (representing 15 *Allium* species) based on 12 biochemical traits including total and reducing sugars, thiosulfinates content, pyruvic acid (Basic, Enzymatically produced and Total ), Total flavonoid content, Total phenol content, and antioxidant activity (DPPH, FRAB), Protein content and carbohydrate percentage. Arrows indicate the direction and contribution of each trait to the variance along the two principal components (Component 1: 37.4%, Component 2: 22.3%).

The principal component analysis (PCA) biplot provides a comprehensive visualization of the biochemical diversity among the evaluated *Allium* species based on twelve key quality traits (**Figure 3**). The first principal component (PC1) explained 37.4% of the total variance, while the second principal component (PC2) accounted for 22.3%, together capturing 59.7% of the total biochemical variation. Red vectors represent individual biochemical traits, with their length and orientation indicating their contribution and correlation to species distribution in the ordination space (**Figure 3**; **Supplementary Figures S2**, **S3**). Traits such as total phenol content, total flavonoid content, carbohydrate percentage, total pyruvic acid, basal pyruvic acid, and FRAP antioxidant activity loaded strongly and positively along PC1, indicating that species positioned in this direction are biochemically enriched in antioxidant-associated and pungency-linked compounds. Conversely, enzymatically produced pyruvic acid showed strong negative loading on PC2, while total sugar, reducing sugar, and allicin/total thiosulfinate content loaded positively along PC2, revealing an inverse association between pungency and sugar/thiosulfinate levels.

*Allium cepa* cultivars such as Bhima Kiran, Bhima Super, and Bhima Shweta, along with *A. ascalonicum*, clustered in the positive PC1 region, reflecting elevated phenolics, flavonoids, and pyruvic acid content. In contrast, *A. sativum* accessions grouped along the positive PC2 axis, indicating higher sugars and thiosulfinates. Wild relatives such as *A. macranthum* and *A. hookeri* were positioned in the negative PC1–PC2 quadrant, suggesting comparatively lower concentrations of the major biochemical traits measured. Species in proximity to a vector direction exhibit higher levels of that trait, while those in opposing directions indicate potential trade-offs or lack of correlation. The PCA pattern highlights three major biochemical groupings: (1) An antioxidant–phenolic–pyruvic cluster (positive PC1), (2) A sugar–thiosulfinate cluster (positive PC2), and (3) A pungency-linked enzymatic pyruvate cluster (negative PC2). These findings underscore the high degree of biochemical diversity within the Allium germplasm, offering valuable insights for breeding programs targeting improved nutritional, medicinal, or flavor-related traits. The separation of species along the two principal components provides a framework for strategic genetic resource utilization and conservation within the genus.

### Principal component contributions

**Figure 4:**
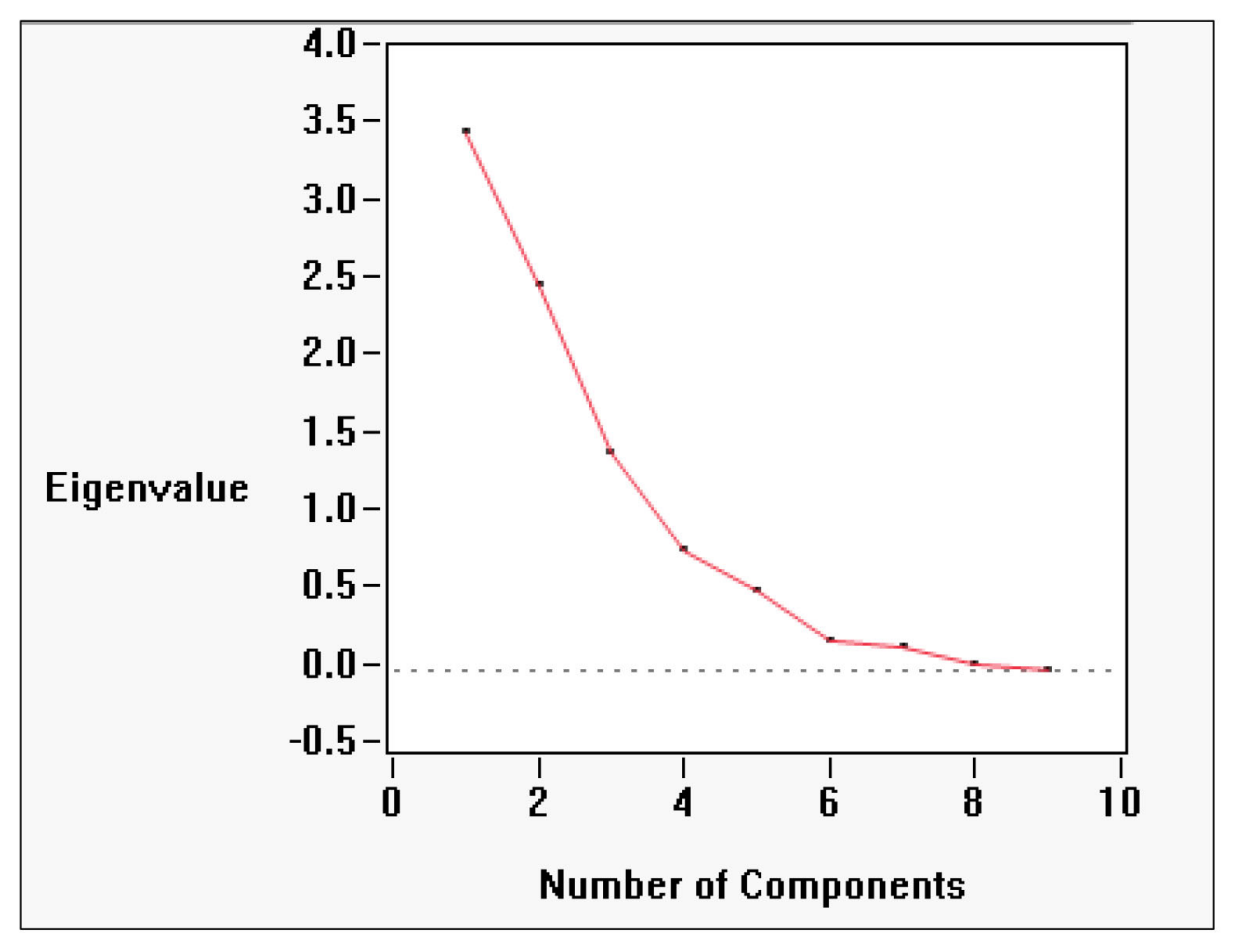
Screen Plot Showing Eigenvalues for Principal Components in *Allium* Biochemical Trait Analysis (Screen plot displaying the eigenvalues associated with each principal component derived from biochemical data of Allium species. The first two components explain the majority of the variance (59.7%) and are typically retained for further analysis).

Principal component analysis (PCA) revealed that biochemical variation in *Allium* germplasm is structured across seven principal components (Prin1–Prin7), with the first three components explaining the majority of the variability (**Table 4**; **Figure 4**). PC1 (Prin1) showed high positive loadings for total flavonoid content (0.8613), antioxidant activity by FRAP (0.5598), total phenol content (0.6676), total pyruvic acid (0.7035), basal pyruvic acid (0.7194), and carbohydrate percentage (0.7574). This axis represents a “phenolic–pyruvic–carbohydrate complex”, indicating genotypes with higher values for these traits also tend to have elevated antioxidant potential and flavor-related compounds. PC2 (Prin2) was dominated by strong positive loadings for total sugar (0.8631), reducing sugar (0.6624), allicin/total thiosulfinate content (0.7166), and basal pyruvic acid (0.5544). This suggests a “sugar–thiosulfinate association**”**, potentially linked to both metabolic energy content and flavor intensity. PC3 (Prin3) was heavily influenced by enzymatically produced pyruvic acid (0.7857) and total pyruvic acid (0.5796), while showing negative association with FRAP antioxidant activity (–0.4974) and reducing sugar (–0.4081). This reflects a “pungency–antioxidant trade-off” dimension, where certain pungency-related compounds increase as some antioxidant metrics decline. PC4 (Prin4) showed moderate loadings for allicin/total thiosulfinate content (0.4924) and protein content (0.3519), but negative associations with carbohydrate percentage (–0.4017) and reducing sugar (–0.4918), representing a “protein–thiosulfinate vs. carbohydrate–sugar” contrast. PC5–PC7 accounted for smaller portions of variability, capturing subtler patterns, such as protein–carbohydrate relationships (PC5) and minor variations in antioxidant measures (PC6, PC7) (**Table 5**; **Supplementary Table S5**).

**Table 4:** Biochemical Parameter Correlation Matrix of 19 *Allium* germplasm representing 15 Allium Species including *A. cepa, A. sativum* and other underutilized species.

Row	Average Allicin TOT Thio (µmol/g FW)	Antioxidant (µmol/g FW) DPPH	Antioxidant (mg/ml)FRAB	Total Flav. Cont. QE (mg/100 g FW)	Total Phenol content GE (mg/100 g FW)	Total Pyruvic Acid (µmol/g FW)	Basal Pyruvic Acid (µmol/g FW)	Enzymatically Produced Pyruvic Acid (µmol/g FW)	Reducing Sugar ( g/100g FW)	Total Sugar ( g/100g FW)	Carbohydrate (%)	Protein (mg/ml)
Average Allicin TOT Thio (µmol/g FW)	1.0000	0.0535	0.0392	0.0740	0.4580	0.5397	0.6671	-0.2147	0.1150	0.3421	0.0959	0.1035
Antioxidant (µmol/g FW) DPPH	0.0535	1.0000	-0.4871	-0.8302	-0.4230	-0.4045	-0.4076	-0.0676	0.3456	0.3733	-0.6868	0.5717
Antioxidant (mg/ml)FRAB	0.0392	-0.4871	1.0000	0.5103	0.4212	-0.0302	0.0368	-0.1705	-0.3208	-0.3714	0.5170	-0.3760
Total Flav. Cont. QE (mg/100 g FW)	0.0740	-0.8302	0.5103	1.0000	0.6504	0.4107	0.4639	-0.0550	-0.2173	-0.2570	0.5759	-0.5793
Total Phenol content GE (mg/100 g FW)	0.4580	-0.4230	0.4212	0.6504	1.0000	0.4016	0.5121	-0.1984	-0.0427	0.2358	0.3528	-0.2481
Total Pyruvic Acid (µmol/g FW)	0.5397	-0.4045	-0.0302	0.4107	0.4016	1.0000	0.9262	0.3681	-0.1855	0.1186	0.4797	-0.3582
Basal Pyruvic Acid (µmol/g FW)	0.6671	-0.4076	0.0368	0.4639	0.5121	0.9262	1.0000	-0.0096	-0.0189	0.2322	0.4475	-0.3659
Enzymatically Produced Pyruvic Acid (µmol/g FW)	-0.2147	-0.0676	-0.1705	-0.0550	-0.1984	0.3681	-0.0096	1.0000	-0.4452	-0.2580	0.1695	-0.0484
Reducing Sugar ( g/100g FW)	0.1150	0.3456	-0.3208	-0.2173	-0.0427	-0.1855	-0.0189	-0.4452	1.0000	0.7969	-0.0020	0.1866
Total Sugar ( g/100g FW)	0.3421	0.3733	-0.3714	-0.2570	0.2358	0.1186	0.2322	-0.2580	0.7969	1.0000	-0.0402	0.2299
Carbohydrate (%)	0.0959	-0.6868	0.5170	0.5759	0.3528	0.4797	0.4475	0.1695	-0.0020	-0.0402	1.0000	-0.4913
Protein (mg/ml)	0.1035	0.5717	-0.3760	-0.5793	-0.2481	-0.3582	-0.3659	-0.0484	0.1866	0.2299	-0.4913	1.0000

(Green– showing positive correlation; Red- showing negative correlation)

**Table 5:** Principal Component Analysis (PCA) Loadings of 12 Biochemical Parameters recorded on 19 *Allium* species representing 15 *Allium* species including *A. cepa*, *A. sativum* and other underutilized species.

Biochemical Traits	Prin1	Prin2	Prin3	Prin4	Prin5	Prin6	Prin7
Average Allicin TOT Thio (µmol/g FW)	**0.3115**	**0.7166**	0.1407	0.4924	0.0655	-0.2455	-0.0301
Antioxidant (µmol/g FW) DPPH	-0.8393	0.2959	0.118	0.1489	0.0633	-0.1227	**0.308**
Antioxidant (mg/ml)FRAB	**0.5598**	-0.3524	-0.4974	**0.2593**	**0.3443**	-0.26	0.1742
Total Flav. Cont. QE (mg/100 g FW)	**0.8613**	-0.1262	-0.2468	-0.0308	-0.1292	**0.2895**	-0.1139
Total Phenol content GE (mg/100 g FW)	**0.6676**	**0.3546**	-0.2827	**0.2871**	0.1329	**0.4251**	0.2224
Total Pyruvic Acid (µmol/g FW)	**0.7035**	**0.3869**	**0.5796**	-0.0426	-0.0424	-0.0416	-0.0075
Basal Pyruvic Acid (µmol/g FW)	**0.7194**	**0.5544**	**0.3047**	0.0467	-0.1889	-0.1158	-0.0764
Enzymatically Produced Pyruvic Acid (µmol/g FW)	0.0922	-0.3415	**0.7857**	-0.2293	**0.3535**	0.1742	0.1689
Reducing Sugar ( g/100g FW)	-0.3211	**0.6624**	-0.4081	-0.4918	0.0412	-0.0428	-0.0766
Total Sugar ( g/100g FW)	-0.1984	**0.8631**	-0.1362	-0.2993	0.1471	0.1468	0.162
Carbohydrate (%)	**0.7574**	-0.0194	-0.084	-0.4017	0.4008	-0.2243	-0.1333
Protein (mg/ml)	-0.666	0.2197	0.0882	0.3519	**0.3986**	0.1985	-0.4102

Bold-major contributing traits in variance from respective component.

Overall, PCA separated the traits into distinct biochemical clusters (i) flavonoid–phenolic–pyruvic–carbohydrate group, (ii) sugar–thiosulfinate group, and (iii) enzymatic pyruvate-driven pungency group highlighting biochemical pathways that may be co-regulated or show metabolic trade-offs. These components can serve as biochemical fingerprints for selecting genotypes with targeted nutritional or flavor profiles.

In summary, the evaluation of 19 *Allium* accessions representing 15 species revealed clear and contrasting patterns of biochemical diversity between cultivated and wild taxa. Cultivated species such as *A. cepa*, *A. sativum*, *A. fistulosum*, and *A. cepa* var. *aggregatum* consistently clustered together, characterized by elevated carbohydrate and sugar contents, a reflection of selective domestication for yield, storability, and consumer preference. In contrast, several wild and underutilized species, notably *A. hookeri* and *A. macranthum*, were enriched in allicin, pyruvic acid, flavonoids, phenolics, and antioxidant activity, emphasizing their importance as reservoirs of nutraceutical compounds and stress-adaptive traits. Similarly, *A. tuberosum* and *A. chinense* displayed distinct organosulfur and antioxidant profiles, while *A. schoenoprasum* and *A. przewalskianum* exhibited elevated antioxidant potential and overall phytochemical richness. Such diversity highlights the evolutionary trade-offs between domestication, which favored palatability and carbohydrate accumulation, and wild relatives, which retained greater phytochemical complexity and adaptive metabolites. Collectively, these findings establish wild *Allium* taxa as valuable genetic and biochemical resources with significant potential for bioprospecting and for breeding onion and garlic cultivars with enhanced nutritional quality, storability, and resilience to biotic and abiotic stresses.

## Discussion

### Phytochemical screening

Phytochemical analysis is a fundamental approach in plant science to identify and characterize bioactive compounds. It reveals the chemical composition of plants, providing insights into metabolic diversity and functional properties. It helps to detect valuable metabolites guides the selection of plant species for further pharmacological studies or natural product development. Germplasm screening for bioactive compounds aids in distinguishing chemotypes, identifying superior accessions, and supporting breeding programs. The phytochemical analysis of various *Allium* species revealed the presence of key bioactive compounds, which align with previous studies on the genus. The phytochemical screening of 19 *Allium* germplasm representing 15 *Allium* species using aqueous and 80% methanol extracts revealed a conserved metabolic profile across the genus, with notable implications for their chemotaxonomic classification and potential bioactive applications. The universal presence of carbohydrates, phenolic compounds, flavonoids, flavonols, and saponins aligns with the well-documented secondary metabolite composition of *Allium* species, which are renowned for their antioxidant, antimicrobial, and anti-inflammatory properties (Lanzotti, 2006).

The presence of carbohydrates in all tested species is expected, as *Allium* plants are known to be rich in polysaccharides such as fructans, which contribute to their nutritional and medicinal value (Seki et al., 2019; Gonzalez-Morales et al., 2017). These compounds have been shown to support gut health and modulate immune responses (Bhat, 2020). The absence of proteins and starch in all tested species is also consistent with previous reports. The absence of proteins and starch may reflect methodological limitations, as methanol extraction is less efficient for polar macromolecules like proteins, which often require aqueous or buffered solvents (Arena et al., 2024; Harborne, 1998). The negative protein tests suggest that the methanolic extract might not effectively extract proteins, or the species naturally have low protein content in their soluble fraction. Starch is generally present in low quantities in *Allium* plants, as their primary storage carbohydrates are fructans rather than starch (Benitez et al., 2017). Similarly, the lack of tannins contrasts with some reports in wild *Allium* species (Mendes et al., 2012), suggesting variability in tannin production across environments or genetic lineages. The uniform absence of alkaloids (confirmed by four distinct tests) further distinguishes *Allium* from alkaloid-rich genera like *Solanum* or *Papaver*, reinforcing its unique chemoprofile (Hesse, 2002).

The widespread presence of phenols, flavonoids, and flavonols aligns with numerous studies highlighting the antioxidant, anti-inflammatory, and antimicrobial properties of *Allium* compounds (Lanzotti, 2006; Benkeblia et al., 2005). These phenolic compounds contribute to the therapeutic potential of *Allium* species and have been associated with reducing the risk of chronic diseases such as cardiovascular disorders and cancer (Najeebullah et al., 2021; Kothari et al., 2020). Phenolics and flavonoids, in particular, are key contributors to the health-promoting effects of *Allium* vegetables, such as garlic (*A. sativum*) and onions (*A. cepa*), and their ubiquitous occurrence in this study underscores their evolutionary significance as defense molecules (Brewster, 2008; Assefa et al., 2018).

Saponins, which were found in all species, are known for their antimicrobial, immune-modulating, and cholesterol-lowering effects (Martins et al., 2016). The detection of steroidal/triterpenoid glycosides *via* Salkowski’s test corroborates earlier studies identifying saponins as hallmark metabolites in *Allium* (Sobolewska et al., 2016). These compounds, such as the immunomodulatory saponins in *A. ampeloprasum*, are linked to antifungal and cholesterol-lowering activities (Lanzotti, 2006). Their presence further supports the medicinal use of *Allium* plants in traditional medicine. In contrast, tannins were absent in all tested species, suggesting a lower astringency compared to other medicinal plants that contain high levels of tannins (Arena et al., 2024; Haslam, 1996). This may indicate that *Allium* species have a different chemical defense mechanism than tannin-rich plants.

The absence of alkaloids, as confirmed by four different tests, is consistent with the general chemical profile of *Allium* species, which are not known for significant alkaloid content (Benkeblia et al., 2005). Instead, sulfur-containing compounds such as allicin, alliin, and ajoene are the primary bioactive compounds in *Allium* species, contributing to their antimicrobial and cardiovascular benefits (Corzo-Martínez et al., 2007; Kothari et al., 2020). The detection of glycosides (positive Salkowski test) suggests the presence of steroidal or terpenoid glycosides, which may contribute to the pharmacological effects of *Allium* species. Glycosides have been linked to various medicinal properties, including cardioprotective and anti-inflammatory activities (Cai et al., 2004). However, the negative results in Keller-Killiani and Borntrager’s tests suggest the absence of cardiac glycosides and anthraquinone glycosides, respectively. However, the negative results for cardiac glycosides (Keller-Killiani) and anthraquinones (Borntrager’s) align with the genus’s limited association with these glycoside subtypes. Finally, the presence of amino acids in all species highlights their potential nutritional benefits, as *Allium* plants are known to contain essential amino acids beneficial for human health (Seki et al., 2019). The amino acid profile of these species may contribute to their role in metabolism and overall dietary value. The reported presence of amino acids under the “fats” category requires clarification, as amino acids are nitrogenous compounds typically associated with proteins. While methanol may extract free amino acids, their classification here likely reflects methodological overlap in colorimetric assays (e.g., ninhydrin reactivity), as noted in biochemical screening protocols (Kumar and Kumar, 2023).

Under methodological considerations the use of 80% methanol likely enhanced the extraction of medium-polarity compounds like flavonoids and saponins while excluding highly polar (e.g., proteins) or nonpolar (e.g., essential oils) metabolites. This aligns with studies advocating methanol’s efficacy for phenolic extraction in *Allium* (Shukla et al., 2024; Arena et al., 2024). However, the absence of alkaloids may reflect solvent incompatibility, as alkaloid isolation often requires acidified solvents.

The uniformity in phytochemical profiles across diverse *Allium* species supports the theory of conserved biosynthetic pathways, possibly driven by ecological pressures such as herbivory or pathogen resistance. For instance, saponins and flavonoids are known deterrents against microbial and insect attacks. These findings reinforce the chemotaxonomic value of flavonoids and saponins in delineating *Allium* subgenera, as proposed by Fritsch and Friesen (2002).

### Analysis of biochemical parameters

The statistical analysis of twelve biochemical parameters in the evaluated *Allium* germplasm revealed marked phenotypic variability, attributable to genetic diversity, environmental influences, and inherent metabolic differences. Coefficient of variation (CV) values indicated traits with the greatest heterogeneity, notably reducing sugar (95.72%) and enzymatically produced pyruvic acid (65.83%), suggesting dynamic post-harvest metabolic changes likely driven by enzymatic activity and carbohydrate–sulfur metabolism interactions. Such variation is consistent with previous findings reporting cultivar- and storage-dependent fluctuations in sugar accumulation and pyruvic acid production (Gonzalez et al., 2017; Benke et al., 2021; Shukla et al., 2024). High mean values of total flavonoid content (23.06 mg 100 g^−1^ FW) and total phenol content (22.22 mg 100 g^−1^ FW) highlight the strong antioxidant potential of the germplasm. These secondary metabolites are widely recognized for their roles in plant defense, stress tolerance, and human health benefits, including anti-inflammatory and free radical scavenging properties (Cai et al., 2004). The broad range observed in total flavonoid content (10.42–48.42 mg 100 g^−1^ FW) and total sugar (0.51–8.79 g 100 g^−1^ FW) suggests that certain genotypes and/or growth conditions enhance secondary metabolite biosynthesis, in line with studies linking flavonoid accumulation to genotype–environment interactions and light-mediated regulation (Jaakola, 2013).

The highest variance (143.66) recorded for total flavonoid content further reinforces its sensitivity to genetic expression and environmental modulation. Similarly, variability in pyruvic acid content aligns with its established role as a biochemical marker of pungency in *Allium* species, where sulfur assimilation and alliinase-mediated reactions are key determinants (Gonzalez-Morales et al., 2017).

Overall, the observed biochemical heterogeneity underscores substantial potential for targeted selection and breeding aimed at enhancing phytochemical richness. Integrating metabolomic profiling with genomic and transcriptomic tools could provide mechanistic insights into the regulation of these traits, enabling the development of *Allium* cultivars with optimized nutritional and functional qualities (Vukovic et al., 2021).

### Cluster analysis

The hierarchical clustering pattern (**Figure 1**) distinctly separated the evaluated *Allium* accessions into two major clusters, reflecting strong underlying biochemical divergence. This grouping was primarily influenced by variability in allicin content, pyruvic acid levels (total, basal, and enzymatic), phenolic and flavonoid concentrations, antioxidant capacity (DPPH and FRAP assays), and sugar composition. Such metabolic differentiation aligns with genetic variation, ecological adaptation, and domestication history within the genus (Kamenetsky and Rabinowitch, 2001; Benke et al., 2024).

Cluster I (red branch in **Figure 1**) predominantly encompassed cultivated bulb-type *A. cepa* forms (common onion, aggregatum) and *A. sativum* (garlic). These accessions were characterized by lower concentrations of allicin, phenolics, and flavonoids, but higher carbohydrate and sugar contents. This pattern reflects domestication-driven selection for milder taste, higher storability, and consumer-preferred sweetness, often at the cost of reduced secondary metabolite abundance (Benkeblia and Lanzotti, 2007). Cluster II (green branch in **Figure 1**) comprised a diverse set of wild and semi-cultivated taxa including *A. fistulosum*, *A. hookeri*, *A. ampeloprasum*, *A. schoenoprasum*, *A. chinense*, and *A. tuberosum* characterized by elevated allicin, pyruvic acid, total phenol, and flavonoid levels, coupled with higher antioxidant activity. These phytochemically rich species align with prior observations (Gonzalez-Morales et al., 2017; Kothari et al., 2020; Shukla et al., 2024) that wild *Allium* possess superior medicinal potential and stress tolerance, likely due to their high investment in defensive secondary metabolites. Pearson’s correlation analysis supported these clustering patterns. Strong positive correlations between total pyruvic acid and basal pyruvic acid (r = 0.9262), and between allicin content and basal pyruvic acid (r = 0.6671), indicate tight co-regulation of pungency-related sulfur compounds—traits that heavily influenced cluster separation. Similarly, moderate correlations between phenolic and flavonoid content (r = 0.6504) and between carbohydrate percentage and FRAP-based antioxidant capacity (r = 0.5170) reflect overlapping biochemical pathways that contribute to trait co-association within clusters. Negative correlations such as between DPPH antioxidant activity and flavonoid content (r = –0.8302) or between carbohydrate percentage and protein content (r = –0.4913) highlight potential metabolic trade-offs. These trade-offs may partly explain why carbohydrate-rich, low-pungency cultivars dominate Cluster I, while phytochemical-dense, high-pungency accessions group in Cluster II.

Overall, the integration of correlation analysis with hierarchical clustering (**Figure 1**) reveals that *Allium* biochemical diversity is structured by strong co-regulation of sulfur metabolism, phenolic pathways, and carbohydrate allocation. This metabolic divergence between domesticated and wild species underscores the breeding potential of wild relatives for enhancing health-promoting phytochemicals, antioxidant capacity, and adaptive resilience in cultivated onions and garlic.

### Principal component analysis and correlation studies

The present study highlights the extensive biochemical diversity within *Allium* species, as revealed by principal component analysis (PCA), aligning with earlier reports that emphasized variability in secondary metabolites, sulfur compounds, and antioxidants across the genus (Kamenetsky and Rabinowitch, 2001; Kumar and Kumar, 2023). The Kaiser–Meyer–Olkin (KMO) statistic (0.65) and a significant Bartlett’s test confirmed the adequacy of the dataset for multivariate reduction, underscoring the robustness of the PCA results. The first two principal components (PC1 and PC2) explained 59.7% of the total biochemical variance, capturing the major axes differentiating cultivated and wild *Allium* germplasm. Key contributors included total sugar, reducing sugar, allicin content, total pyruvic acid, phenolic content, and antioxidant activity—traits also emphasized in interspecific diversity studies by Jikah et al. (2024) and Havey (2018). Similar analyses have demonstrated that PCA effectively distinguishes garlic (*A. sativum*) and onion (*A. cepa*) accessions on the basis of their biochemical fingerprints (Maccelli et al., 2020). The clustering observed in the dendrogram was consistent with PCA groupings, reinforcing the idea that biochemical composition serves as both a taxonomic marker and a functional discriminator in *Allium* species (Kamenetsky and Rabinowitch, 2001).

The PCA biplot (**Figure 3**) revealed three major biochemical clusters. The first, an antioxidant–phenolic–pyruvic module represented along PC1, included traits such as total phenol, flavonoid content, carbohydrate percentage, and pyruvic acid indices. This grouping indicates that phenolic metabolism and pungency traits are co-regulated, consistent with reports that phenolics and sulfur-derived pyruvates share overlapping biosynthetic pathways contributing to both antioxidant potential and flavor intensity (Benke et al., 2024). Pearson’s correlation supported this interpretation, showing strong positive associations between total pyruvic acid and basal pyruvic acid (r = 0.9262) and between allicin and basal pyruvic acid (r = 0.6671). These correlations confirm the biochemical link between pungency-related compounds and sulfur metabolism. Cultivated *A. cepa* genotypes (e.g., Bhima Kiran, Bhima Super, Bhima Shweta) and *A. ascalonicum* were positioned positively along this axis, reflecting their higher phenolic and pyruvic acid concentrations, which enhance both health-promoting properties and characteristic onion pungency (Martins et al., 2016).

The second grouping, a sugar–thiosulfinate module aligned with PC2, was dominated by strong loadings from total sugar, reducing sugar, and allicin/total thiosulfinate content. *A. sativum* accessions clustered here, reflecting their elevated thiosulfinate levels and carbohydrate richness—biochemical features long recognized for contributing to garlic’s medicinal efficacy and storability (Benkeblia and Lanzotti, 2007). Pearson’s correlation further supported this association, as total phenol content was moderately correlated with total flavonoid content (r = 0.6504) and basal pyruvic acid (r = 0.5121), suggesting that phenolic metabolism overlaps with sulfur pathways. The close association between sugars and thiosulfinates highlights coordinated regulation of primary and secondary metabolism, whereby carbohydrate flux supports the biosynthesis of organosulfur compounds (Khar et al., 2022). These biochemical linkages explain why sugars influence both taste and postharvest behavior in *Allium* crops (Benkeblia and Shiomi, 2006; Rabinowitch and Currah, 2002).

A third cluster, driven by PC3 contributions, reflected a pungency–antioxidant inverse relationship. Enzymatically produced pyruvic acid was negatively associated with FRAP-based antioxidant capacity and reducing sugars. Pearson’s correlations confirmed these antagonisms: reducing sugar was negatively correlated with enzymatic pyruvic acid (r = –0.4452), while FRAP activity was negatively correlated with DPPH scavenging capacity (r = –0.4871). Similarly, DPPH-based antioxidant activity was strongly negatively correlated with total flavonoid content (r = –0.8302) and carbohydrate percentage (r = –0.6868). These results suggest that radical scavenging efficiency in *Allium* is supported by non-flavonoid antioxidants, particularly organosulfur derivatives, which contribute to pungency and antimicrobial activity but do not always act as direct antioxidants (Benkeblia et al., 2005; Seki et al., 2019). The observed inverse associations underscore evolutionary metabolic balancing: selection for pungency in wild relatives often coincides with enhanced sulfur metabolism but lower sugar- or flavonoid-derived antioxidant activity. Conversely, cultivated types exhibit greater carbohydrate accumulation but relatively diluted phytochemical richness. Wild species such as *A. macranthum* and *A. hookeri* occupied the negative quadrants of the PCA space, reflecting unique but lower trait concentrations, likely adaptations to ecological niches (Corzo-Martínez et al., 2007; Kamenetsky and Rabinowitch, 2001).

Overall, the PCA and correlation analyses clearly separate domesticated from wild *Allium* germplasm. Cultivated onions and shallots were enriched in phenolic and pyruvic acid traits, while garlic accessions showed sugar–thiosulfinate dominance. Wild and underutilized taxa, although lower in trait concentrations, represent reservoirs of alleles for stress resilience, pungency, and medicinal properties (Fritsch and Friesen, 2002). The scree plot confirmed that the first three components capture most of the meaningful biochemical variance, supporting the interpretation of three primary axes: (i) phenolic–pyruvic–carbohydrate complex, (ii) sugar–thiosulfinate association, and (iii) enzymatic pyruvate–antioxidant inverse association. Together, these axes reflect distinct metabolic fingerprints within the *Allium* genus (Havey, 2018).

In summary, *Allium* biochemical diversity is structured along metabolic pathways that reveal evolutionary balances between primary (sugars, carbohydrates) and secondary (phenolics, flavonoids, organosulfur compounds) metabolism. Integrating PCA and correlation networks provides a framework for exploiting this diversity in breeding programs balancing flavor, nutritional quality, and health-promoting properties while also guiding the conservation of wild relatives as reservoirs of unique metabolic traits.

## Conclusion

This study demonstrates that *Allium* species harbor a consistent yet diverse repertoire of phytochemicals, underscoring their importance as promising nutraceutical resources. Comparative profiling revealed marked biochemical divergence between cultivated onions and garlic, which clustered together with higher carbohydrate and sugar contents, and their wild and underutilized counterparts such as *A. hookeri* and *A. macranthum*, which were enriched in allicin, pyruvic acid, flavonoids, phenolics, and antioxidant activity. Correlation analyses integrated with PCA loadings further highlighted strong positive associations among phenolics, flavonoids, and pyruvic acid, while sugars exhibited antagonistic relationships with pungency-related metabolites. These trends reflect evolutionary trade-offs between domestication for yield and palatability versus the retention of stress-responsive and health-promoting phytochemicals in wild taxa.

Collectively, the findings establish wild *Allium* species as reservoirs of bioactive compounds and stress-resilient alleles, offering untapped potential for nutraceutical exploitation and genetic improvement. By linking biochemical diversity with evolutionary and agronomic contexts, this work provides a robust framework for targeted bioprospecting and for breeding programs aimed at enhancing nutritional quality, storability, and resilience in onion and garlic. Future research should integrate advanced metabolomics platforms (e.g., LC–MS, HPLC-based flavonoid quantification) with functional bioassays to validate therapeutic relevance and to accelerate the translation of phytochemical diversity into consumer-oriented, climate-resilient cultivars.

## Future perspectives

The biochemical diversity revealed by PCA and correlation analyses has far-reaching implications for crop improvement, functional food innovation, and pharmacological research. The clustering of high-antioxidant species offers guidance for breeding programs targeting enhanced nutritional quality, while *Allium* species enriched in allicin and pyruvic acid provide promising avenues for novel nutraceutical and medicinal formulations (Borlinghaus et al., 2014).

Future studies that integrate genomic, metabolomic, and environmental data will be essential to unravel the biochemical plasticity of *Allium* species and its relevance to human health and agricultural sustainability (Kamenetsky and Rabinowitch, 2001). Wild relatives, with their superior levels of bioactive compounds, represent invaluable genetic reservoirs for improving disease resistance, abiotic stress tolerance, and nutritional properties in cultivated onion and garlic (McCallum et al., 2006; Benke et al., 2024). Advanced breeding strategies including marker-assisted selection (MAS) and introgression of wild alleles could accelerate the incorporation of health-promoting traits into commercial cultivars (Khar et al., 2022). Harnessing this biochemical diversity will be pivotal for developing resilient, nutrient-dense *Allium* varieties that balance consumer preferences with nutritional security and climate resilience.

## Data Availability

The original contributions presented in the study are included in the article/**Supplementary Material**. Further inquiries can be directed to the corresponding author.
