# Supplementary material for: Biochemical diversity in Allium species: key metabolite profiles for breeding and bioprospecting
Source: Front Plant Sci. 2025 Nov 5;16:1618572. doi: 10.3389/fpls.2025.1618572 (PMC12627065; doi:10.3389/fpls.2025.1618572)
Supplement: Supplementary file 1 [file DataSheet1.docx]

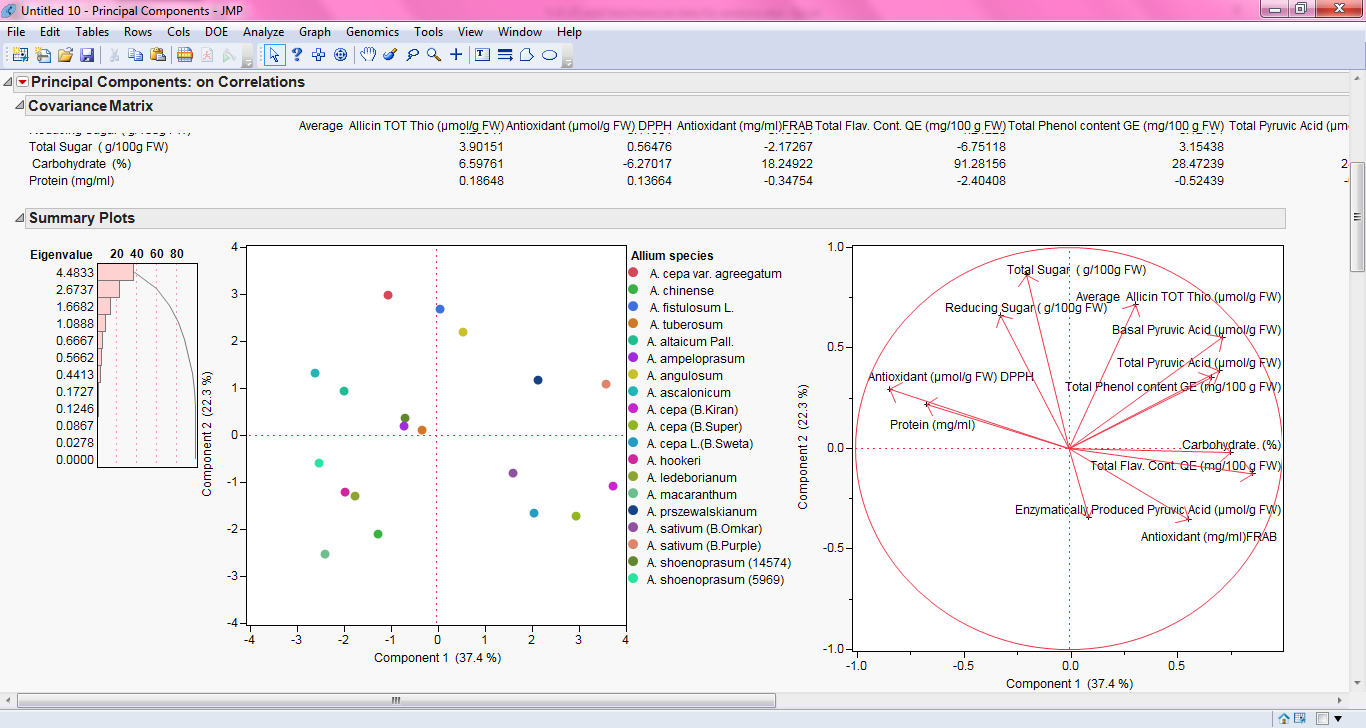


Figure S1: Principal Component Analysis (PCA) of 15 *Allium* Species (19 germplasm) Based on Biochemical Traits


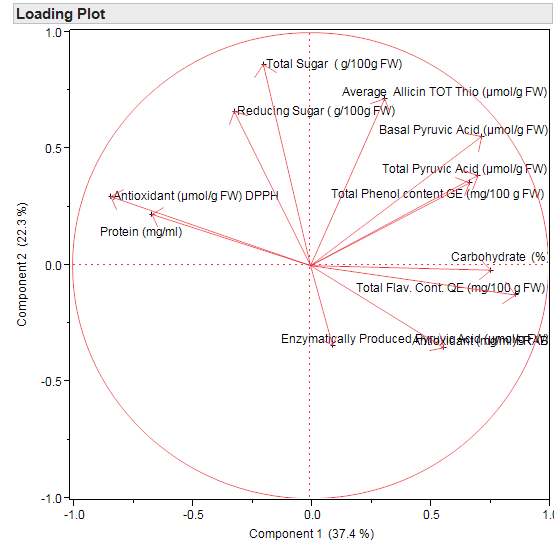


Figure S2: Loading plot of principal components showing contribution of biochemical traits in *Allium* germplasm (19)
