# Supplementary material for: Biochemical diversity in Allium species: key metabolite profiles for breeding and bioprospecting
Source: Front Plant Sci. 2025 Nov 5;16:1618572. doi: 10.3389/fpls.2025.1618572 (PMC12627065; doi:10.3389/fpls.2025.1618572)
Supplement: Supplementary file 2 [file Table1.docx]

Table S1: Phytochemcial analysis of 19 *Allium* germplasm (representing 15 *Allium* species) using juice extractions in distilled water

| **Sr. No.** | *Allium* species | *A. fistulosum* | *A. cepa agreegatum* | *A. prszewalskianum* | *A. hookeri* | *A. shoeprasum* | *A. altaicum pall* | *A. ledeborianum* | *A. chinense* | *A. macranthum* | *A. shoenoprasum* | *A. ampeloprasum* | *A. angulosum* | *A. tuberosum* | *Allium ascalonicum* | *A. sativum* var. Bhima Omkar | *A. sativum* var. Bhima Purple | *A. cepa var.* Bhima Super | *A. cepa var.* Bhima Shweta | *A.cepa var. Bhima Kiran* |
| --- | --- | --- | --- | --- | --- | --- | --- | --- | --- | --- | --- | --- | --- | --- | --- | --- | --- | --- | --- | --- |
| 1 | Carbohydrate | Y | Y | Y | Y | Y | Y | Y | Y | Y | Y | Y | Y | Y | Y | Y | Y | Y | Y | Y |
| 2 | Protein | Not applicable | | | | | | | | | | | | | | | | | | |
| 3 | Starch | X | X | X | X | X | X | X | X | X | X | X | X | X | X | X | X | X | X | X |
| 4 | Phenol | Y | Y | Y | Y | Y | Y | Y | Y | Y | Y | Y | Y | Y | Y | Y | Y | Y | Y | Y |
| 5 | Flavonoids and flavonol | Y | Y | Y | X | Y | Y | Y | Y | Y | Y | Y | X | X | X | Y | X | X | X | Y |
| 6 | Tanin | Not applicable | | | | | | | | | | | | | | | | | | |
| 7 | Saponin | Not applicable | | | | | | | | | | | | | | | | | | |
| 8 | Anthoquinon | Not applicable | | | | | | | | | | | | | | | | | | |
| 9 | Alkaloids | Not applicable | | | | | | | | | | | | | | | | | | |
|  | 1. Mayers method | X | X | X | X | X | X | X | X | X | X | X | X | X | X | X | X | X | X | X |
|  | 2. Dragondorf | X | X | X | X | X | X | X | X | X | X | X | X | X | X | X | X | X | X | X |
|  | 3. Wagners | X | X | X | X | X | X | X | X | X | X | X | X | X | X | X | X | X | X | X |
|  | 4. Hagers | X | X | X | X | X | X | X | X | X | X | X | X | X | X | X | X | X | X | X |
| 10 | Glycosides |  |  |  |  |  |  |  |  |  |  |  |  |  |  |  |  |  |  |  |
|  | 1. Keller-Killiani | Y | Y | Y | Y | Y | Y | Y | Y | Y | Y | Y | Y | Y | Y | Y | Y | Y | Y | Y |
|  | 2. Borntragers | X | X | X | X | X | X | X | X | X | X | X | X | X | X | X | X | X | X | X |
|  | 3. salkowskis | Y | Y | Y | Y | Y | Y | Y | Y | Y | Y | Y | Y | Y | Y | Y | Y | Y | Y | Y |
| 11 | Fats |  |  |  |  |  |  |  |  |  |  |  |  |  |  |  |  |  |  |  |
|  | 1. Steroids | Y | Y | Y | Y | Y | Y | Y | Y | Y | Y | Y | Y | Y | Y | Y | Y | Y | Y | Y |
|  | 2. Amino acids (Ninhydrin test) | Y | Y | Y | Y | Y | Y | Y | Y | Y | Y | Y | Y | Y | Y | Y | Y | Y | Y | Y |
