# Supplementary material for: Biochemical diversity in Allium species: key metabolite profiles for breeding and bioprospecting
Source: Front Plant Sci. 2025 Nov 5;16:1618572. doi: 10.3389/fpls.2025.1618572 (PMC12627065; doi:10.3389/fpls.2025.1618572)
Supplement: Supplementary file 4 [file Table3.docx]

Table S3 : Hierarchical clustering of 15 *Allium* species (19 Allium germplasm) based on genetic distance

| **Number of Clusters** | **Distance** | **Leader** | **Joiner** |
| --- | --- | --- | --- |
| 18 | 1.215639 | *A. fistulosum L.* | *A. angulosum* |
| 17 | 1.421882 | *A. shoenoprasum (14574)* | *A. tuberosum* |
| 16 | 1.627688 | *A. cepa L.(*Bhima Shweta*)* | *A. cepa (*Bhima.Super*)* |
| 15 | 1.768784 | *A. chinense* | *A. macaranthum* |
| 14 | 1.800497 | *A. ledeborianum* | *A. shoenoprasum (5969)* |
| 13 | 1.823046 | *A. altaicum Pall.* | *A. ampeloprasum* |
| 12 | 1.96428 | *A. cepa var. agreegatum* | *A. ascalonicum* |
| 11 | 2.466682 | *A. shoenoprasum (14574)* | *A. sativum (Bhima Omkar)* |
| 10 | 2.549795 | *A. ledeborianum* | *A. chinense* |
| 9 | 2.720746 | *A. prszewalskianum* | *A. sativum (*Bhima Purple*)* |
| 8 | 2.758415 | *A. cepa L.(*Bhima Shweta*)* | *A. cepa (*Bhima Kiran*)* |
| 7 | 2.946083 | *A. fistulosum L.* | *A. shoenoprasum (14574)* |
| 6 | 3.357143 | *A. hookeri* | *A. ledeborianum* |
| 5 | 3.540344 | *A. fistulosum L.* | *A. altaicum Pall.* |
| 4 | 4.202878 | *A. prszewalskianum* | *A. cepa L.(Bhima Sweta)* |
| 3 | 4.968762 | *A. fistulosum L.* | *A. cepa var. agreegatum* |
| 2 | 5.579991 | *A. fistulosum L.* | *A. hookeri* |
| 1 | 7.92014 | *A. fistulosum L.* | *A. prszewalskianum* |

Table S4: Hierarchical clustering of 12 biochemical traits based on genetic distance (Ward, 1963 method)

| **Number of Clusters** | **Distance** | **Leader** | **Joiner** |
| --- | --- | --- | --- |
| 11 | 1.152473 | Total Pyruvic Acid (µmol/g FW) | Basal Pyruvic Acid (µmol/g FW) |
| 10 | 1.911828 | Reducing Sugar ( g/100g FW) | Total Sugar ( g/100g FW) |
| 9 | 2.5084 | Total Flav. Cont. QE (mg/100 g FW) | Total Phenol content GE (mg/100 g FW) |
| 8 | 2.77674 | Antioxidant (µmol/g FW) DPPH | Protein (mg/ml) |
| 7 | 2.948472 | Antioxidant (mg/ml)FRAB | Carbohydrate (%) |
| 6 | 3.012658 | Average Allicin TOT Thio (µmol/g FW) | Total Pyruvic Acid (µmol/g FW) |
| 5 | 3.430024 | Antioxidant (mg/ml)FRAB | Total Flav. Cont. QE (mg/100 g FW) |
| 4 | 4.483286 | Antioxidant (µmol/g FW) DPPH | Reducing Sugar ( g/100g FW) |
| 3 | 4.806779 | Average Allicin TOT Thiosulphinate content (µmol/g FW) | Enzymatically Produced Pyruvic Acid (µmol/g FW) |
| 2 | 5.269087 | Average Allicin TOT Thiosulphinate content (µmol/g FW) | Antioxidant (mg/ml)FRAB |
| 1 | 8.116626 | Average Allicin TOT Thiosulphinate content (µmol/g FW) | Antioxidant (µmol/g FW) DPPH |
