# Supplementary material for: Biochemical diversity in Allium species: key metabolite profiles for breeding and bioprospecting
Source: Front Plant Sci. 2025 Nov 5;16:1618572. doi: 10.3389/fpls.2025.1618572 (PMC12627065; doi:10.3389/fpls.2025.1618572)
Supplement: Supplementary file 5 [file Table5.docx]

Table S5: Principal Component Analysis Results Showing Eigenvalues, Percentage Variance, Cumulative Variance, and Chi-Square Statistics of 19 *Allium* germplasm representing 15 species on the basis of 12 biochemical traits

| Number | Eigenvalue | Percent | Cum Percent | ChiSquare | DF | Prob>ChiSq |
| --- | --- | --- | --- | --- | --- | --- |
| 1 | 4.4833 | 37.361 | 37.361 | 330.754 | 65.298 | <.0001 |
| 2 | 2.6737 | 22.281 | 59.642 | 278.9 | 61.202 | <.0001 |
| 3 | 1.6682 | 13.902 | 73.544 | 238.452 | 54.168 | <.0001 |
| 4 | 1.0888 | 9.073 | 82.617 | 206.408 | 45.923 | <.0001 |
| 5 | 0.6667 | 5.556 | 88.173 | 180.256 | 37.646 | <.0001 |
| 6 | 0.5662 | 4.719 | 92.891 | 160.9 | 29.505 | <.0001 |
| 7 | 0.4413 | 3.677 | 96.569 | 139.787 | 22.428 | <.0001 |
| 8 | 0.1727 | 1.439 | 98.008 | 115.445 | 15.956 | <.0001 |
| 9 | 0.1246 | 1.038 | 99.046 | 102.56 | 10.26 | <.0001 |
| 10 | 0.0867 | 0.722 | 99.768 | 89.831 | 5.853 | <.0001 |
| 11 | 0.0278 | 0.232 | 100 | 73.517 | 2.4 | <.0001 |
| 12 | 0 | 0 | 100 | 0 |  |  |
